# Patient preferences for generic substitution policies: a discrete choice experiment in China

**DOI:** 10.3389/fphar.2024.1400156

**Published:** 2024-07-02

**Authors:** Lingli Zhang, Dashuang Li, Xin Li, Jianzhou Yan

**Affiliations:** ^1^ School of International Pharmaceutical Business, China Pharmaceutical University, Nanjing, China; ^2^ Medical Services Department, Qilu Hospital of Shandong University, Jinan, China; ^3^ School of Pharmacy, Nanjing Medical University, Nanjing, China; ^4^ School of Health Policy and Management, Nanjing Medical University, Nanjing, China; ^5^ Center for Global Health, School of Public Health, Nanjing Medical University, Nanjing, China; ^6^ Research Center of National Drug Policy and Ecosystem, China Pharmaceutical University, Nanjing, China

**Keywords:** generic substitution, patient preferences, discrete choice experiment, generic consistency evaluation, China

## Abstract

**Background:** Generic substitution policies have been widely implemented worldwide to enhance the accessibility of medications. Nevertheless, certain patients have voiced discontent with these policies. This study aimed to evaluate the patient preferences for generic substitution policies and explore the potential for optimization to enhance patient acceptance.

**Methods:** A discrete choice experiment (DCE) was conducted to estimate the relative importance (RI) of five attributes, including generic consistency evaluation (GCE), reimbursement rate, medication use control, information disclosure, and post-marketing surveillance. Respondents were recruited among inpatients and outpatients in three cities and surveys were conducted face-to-face. Preference coefficients, RI of attributes, and the uptake rate of various policies were computed using a mixed logit model. The interaction effects were also included to examine preference heterogeneity.

**Results:** A total of 302 patients completed the survey. All five attributes significantly impacted policy acceptance. GCE held the highest RI value at 56.64%, followed by reimbursement rate (RI = 12.62%), information disclosure (RI = 12.41%), post-marketing surveillance (RI = 9.54%), and medication use control (RI = 8.80%). Patient preferences varied depending on their gender and income. The patient uptake rate of China’s current policy was only 68.56%. If all generics were to pass GCE without altering the other attributes, the uptake rate of policies would rise to 82.63%. Similarly, implementing information disclosure without changing other attributes would result in a 78.67% uptake rate, which is comparable to the effect of a 10% increase in reimbursement rate for generics (78.81%). Combining these policies could mitigate the adverse effects of mandatory substitution on patient.

**Conclusion:** Chinese patient preferences for generic substitution policies were mainly influenced by GCE. China’s current generic substitution policy has room for further optimization to enhance patient acceptance.

## 1 Introduction

Generic substitution involves replacing a brand-name medication with a generic version containing the same active ingredient. Unlike brand-name drugs, generics are authorized based on demonstrating bioequivalence to the reference drug, circumventing the need for extensive clinical trials (Di Paolo and Arrigoni, 2018). Generic drugs are typically priced lower than their brand-name counterparts. The introduction of new generic drugs in the United States between 2018 and 2020 saved approximately $53.3 billion within the first year of approval (Conrad et al., 2022). Due to their cost-effectiveness, generic substitution is recognized as a means to enhance medication affordability and accessibility (Hassali et al., 2014; El-Harakeh and Haley, 2022). Moreover, increased use of affordable generics can improve long-term treatment adherence and clinical outcomes (Choudhry et al., 2016).

Policymakers and payers have long aimed to promote generic substitution. Over the years, various strategies have been employed to incentivize this practice, including mandatory or permissive substitution, higher co-payments for brand-name drugs, and providing information and education to healthcare professionals and patients (Wouters et al., 2017; Howard et al., 2018; Sacks et al., 2021). Furthermore, complementary policies are essential to address potential barriers that may hinder generic substitution adoption. For instance, drug regulatory authorities should ensure the bioequivalence of generics and establish a post-marketing surveillance and monitoring system to detect and address potential quality issues (Hassali et al., 2014).

In the Chinese healthcare system, physicians commonly prescribe medicines using generic names and select a specific product based on manufacturing company or drug price information (Zhao et al., 2021). China previously lacked a comprehensive national policy advocating for the use of generic drugs (Sun, 2013). However, in recent years, the country has taken steps to encourage generic substitution, primarily through two significant policies: generic consistency evaluation (GCE) since 2016 and national volume-based drug procurement (NVBP) since 2019. GCE’s main goal was to enhance the quality of generic medicines. Previously, generics only needed to demonstrate bioequivalence to other marketed drugs, including generics. However, GCE required manufacturers to demonstrate interchangeability between generic drugs and their originator counterparts (General Office of the State Council of China, 2016). Successfully passing GCE results in a certification label on the generic drug’s packaging, making it easily identifiable to the public. Additionally, the government has implemented measures to incentivize manufacturers to conduct GCE. For example, only generics that pass GCE are eligible to participate in the NVBP alongside original drugs. Generics failing to meet GCE criteria may gradually be withdrawn from the market. NVBP involves bidding and pricing based on procurement volume, with public healthcare providers facing penalties if they fail to meet promised procurement volumes to protect the rights of the winning manufacturer. Generic drugs typically dominate the bidding process due to their lower prices, establishing market dominance (Yuan et al., 2021).

Despite the potential cost savings, reduced blood concentrations and lack of therapeutic equivalence have been observed in real-world settings in China following the generic substitution (Zhang et al., 2023). Furthermore, some patients express dissatisfaction with generic substitution policies, believing they should have the right to choose between generics and originator drugs (El-Dahiyat and Kayyali, 2013; Granlund and Sundström, 2018). Since patients ultimately have the authority to accept generics, it is crucial to understand their preferences regarding generic substitution policies.

The discrete choice experiment (DCE) is a quantitative method used to elicit individual stated preferences by presenting various alternatives with distinct attribute levels (Lancsar and Louviere, 2008). Several studies have used DCE to examine preferences for health policies (Chandoevwit and Wasi, 2020; Obadha et al., 2020; Geng et al., 2022; Mouter et al., 2022; Nicolet et al., 2022). However, to our knowledge, no study has explored patient preferences for policies promoting generic use and investigated patient support for a wide range of generic substitution policies using DCE.

Therefore, this study aims to assess patient preferences for generic substitution policies using DCE and predict the uptake rate of various policies. The results will provide evidence for the development and optimization of generic substitution policies guided by patient preferences.

## 2 Methods

We detail the methodology employed in this study, following the recommended research practices of the International Society for Pharmacoeconomics and Outcomes Research (ISPOR) (Bridges et al., 2011). The key steps include defining the research question, identifying attributes and levels, constructing choice tasks, collecting data, and analyzing data.

### 2.1 Identifying attributes and levels

To identify the attributes and levels for the DCE, we utilized a multi-faceted approach involving a literature review, feedback from focus group discussions, and expert opinions.

To begin, a comprehensive literature review was conducted to identify potential policies for promoting generic drug usage. We searched English-language articles published between 1 January 2002, and 31 July 2022, on PubMed and Web of Science using search terms such as “generic medicine,” “generic drug,” “generic substitution,” “drug substitutions,” “policy,” “intervention,” “promotion,” and “incentive” in the title or abstract. Several narrative reviews provided insights into various generic policies worldwide. For instance, Kaplan et al. (2012) identified policies related to pricing, competition, prescribing, dispensing, trade/patents, and reimbursement. Babar et al. (2014) summarized seven main themes, including education, financial incentives, advertising, free generic medicine trials, administrative forms, electronic prescribing, and medicines use review. Hassali et al. (2014) reviewed experiences in promoting generic substitution in eight countries, highlighting common policies like mandatory generic substitution and generic prescribing. Thus, we identified eight candidate attributes (Supplementary Table S1) from the literature review, after consolidating similar attributes and eliminating those that were irrelevant to patient.

Additionally, a convenience sample of 20 patients from a local hospital participated in a focus group discussion. The discussion focused on the polices that influenced their choice between generics and original medicines. Patients expressed concerns regarding GCE, post-marketing surveillance in actual use, price, reimbursement, and prescription. We added GCE and post-marketing surveillance to the candidate attributes list based on the patient discussion. Combining insights from the literature review and patient discussions, we identified 10 candidate attributes and their corresponding levels.

We subsequently conducted in-depth interviews with five professionals in pharmaceutical administration and five experts with experience in DCE. The interviews lasted on average 34 min. During these interviews, the experts provided feedback on candidate attributes and levels, assessed the feasibility of implementing policies in China, and determined the appropriateness of DCE incorporation (Supplementary Table S2). We removed and adjusted some attributes according to the experts’ opinions.

The levels for each attribute in our DCE were determined through a comprehensive review of existing literature and focus group discussions with patients. In addition, expert consultation was used to validate that the level of each attribute was realistic and meaningful.

Ultimately, we selected five attributes to describe potential generic policies in China: GCE, reimbursement rate, medicine use control, information disclosure, and post-marketing surveillance. Four of these attributes had three levels, while one had two levels. Table 1 outlines the attributes and their levels.

**TABLE 1:** Attributes and levels in the discrete choice experiment.

Attribute	Level	Definition
Generic consistency evaluation	None	No implementation of generic consistency evaluation, meaning no knowledge of the similarity in safety and efficacy of generics to brand-name drugs
	Some	Some generics pass consistency evaluation compared to brand-name drugs, while others are marketed with only proven the similarity to other generics
	All	All generics pass consistency evaluation and demonstrate the similarity in safety and efficacy to brand-name drugs
Reimbursement rate	Same	Same reimbursement rate for generics and brand-name drugs
	5% higher	5% higher reimbursement rate for generics compared to brand-name drugs
	10% higher	10% higher reimbursement rate for generics compared to brand-name drugs
Medicine use control	Mandatory	Mandatory substitution of generic drugs for brand-name drugs
	Priority	Giving priority to the use of generic drugs over brand-name drugs
	Autonomy	Patients have autonomy in selecting between generic and brand-name drugs
Information disclosure	No	Information on generic drugs is not widely and publicly available
	Yes	Information on generic drugs is widely and publicly available
Post-marketing surveillance	None	No post-marketing surveillance of the efficacy and safety of generics compared to the brand-name drugs
	Some	Surveillance of the efficacy and safety of some post-marketing generic drugs with high risk compared to the brand-name drugs
	All	Surveillance of the efficacy and safety of all post-marketing generic drugs compared to the brand-name drugs

### 2.2 Experiment design

To construct the choice tasks, we employed a D-efficient design using JMP Pro 14 software. Parameters for this design (Supplementary Table S3) were determined based on the patient opinions in focus group discussions. For instance, higher numbers of drugs passing GCE, higher reimbursement rates, less medicine use control, increased disclosure, and enhanced post-market surveillance were generally preferred. Each choice task consisted of two options, with variations in levels across five attributes. Considering the widespread implementation of generic substitution policies and the primary aim of the study being the comparison of various policies, we did not include an opt-out option (Veldwijk et al., 2014; Campbell and Erdem, 2019). The choice tasks were presented to respondents without labels to ensure attention to attributes. A total of 16 choice tasks were generated and divided into two blocks, with each block containing eight choice tasks. An example of a choice task was displayed in Table 2. Attribute order in the choice tasks was randomly presented to respondents to mitigate potential order effects.

**TABLE 2:** A choice task example.

	Policy A	Policy B
Generic consistency evaluation	Some	All
Reimbursement rate	5% higher	10% higher
Medicine use control	Autonomy	Mandatory
Information disclosure	No	Yes
Post-marketing surveillance	Some	All
Which one do you prefer?	□	□

In addition to the choice tasks, the questionnaire included attribute and level definitions and comprehension test, sociodemographic inquiries, and a “quality control” choice task where policy A was clearly superior to policy B. Given that patient preferences might differ from our perception of the dominant option, we conducted the main analysis using questionnaires that chose the dominant option, while also incorporating questionnaires that did not select the dominant option for the sensitivity analysis. Before the formal survey, a pilot study was conducted with 30 patients at a local hospital to assess questionnaire comprehensibility and incorporate feedback.

### 2.3 Survey

The sample size was determined using Orme’s formula [N > 500*c/(t*a)], which required a minimum of 94 respondents (with a maximum of three levels, eight choice tasks, and two alternatives). To ensure a broad representation of geographic and economic conditions, we selected three cities-Nanjing, Zhengzhou, and Xi’an-from different regions of China: eastern, central, and western. In each city, we selected a large comprehensive hospital that serves a significant portion of the local population and has a diverse patient base. We also considered the accessibility of the hospitals to our research team and the willingness of the hospitals to participate in the study. We aimed to collect 100 questionnaires from each hospital, resulting in a total sample size of 300. A convenience sampling approach was used to recruit inpatients and outpatients in the selected hospitals. Sampling incorporated predetermined quotas for gender, age, and department visited (Supplementary Table S4) to enhance sample representativeness. Eligible respondents were aged 18–60 years and had used medication within the past 12 months. Trained investigators provided face-to-face explanations of the survey’s purpose, basic information about generics, and instructions on questionnaire completion. All questionnaires were paper-based and self-administered. Respondents who completed the survey received a gift worth $3. Data collection took place from February to April 2023, with informed consent obtained at the survey’s outset. The study was approved by the Ethics Committee of China Pharmaceutical University (CPU2022117) and written informed consent was obtained from all individual participants.

Questionnaires were excluded based on three criteria: 1) completion time less than 2 min, which is less than one-third of the median time in the pilot survey; 2) failed the comprehension test; 3) consistently selecting the same answer for each choice (i.e., consistently choosing either the left or right option).

### 2.4 Statistical analysis

Data were analyzed using a mixed logit model to determine attribute level utilities and relative importance (RI). All attributes were treated as categorical variables and dummy-coded, with an alternative-specific constant included. Random parameters were estimated using 500 standard Halton draws, which achieved stable model estimates. The mean coefficient represented average preference values for attribute levels, while the standard deviation (SD) indicated preference heterogeneity among patients. RI for each attribute was calculated by dividing the difference between coefficients for the best and worst levels by the sum of all attribute differences (Lancsar et al., 2007). Interaction effects between the DCE attributes and respondent characteristics were also included to account for preference heterogeneity.

In the scenario simulation analysis, we estimated the uptake rate of different scenarios using mixed logit coefficients. Uptake rate represented the probability of patient acceptance for the policy, illustrating how policy optimization could incentivize patient support. The current generic policy in China, involving some generics passing GCE, the same reimbursement rate for generics and brand-name drugs, priority for generics, lack of information disclosure, and no post-marketing surveillance, served as a base scenario. Hypothetical scenarios with improved attributes compared to the base scenario were developed, and uptake rates of scenarios were estimated. 95% confidence intervals (CI) were calculated using the bootstrap method. Statistical analysis was performed using Stata software (version 16).

## 3 Results

### 3.1 Respondent characteristics

A total of 302 patients completed the questionnaire. No significant differences were found among the quotas and patients who completed the questionnaire (Supplementary Table S4). Out of these, 25 questionnaires were excluded due to completion time less than 2 min, 12 were excluded for failing the comprehension test, 23 were excluded for selecting the same answer for each choice, and 31 were excluded for not selecting the dominant option. Finally, 211 respondents were included in the main analysis, distributed across the cities of Nanjing (75 respondents), Zhengzhou (69 respondents), and Xi’an (67 respondents). Of the 211 participants, 46.92% were male, 37.44% were aged between 30 and 44, 34.12% reported an annual income ranging from 30,000 to 80,000 Chinese Yuan (CNY), and 67.77% had Urban Employees Basic Medical Insurance. Sociodemographic characteristics of the respondents are presented in Table 3.

**TABLE 3:** Sociodemographic of respondents included in the analysis.

Characteristic	Patients (n = 211)	
	Number	Percentage (%)
Gender		
Male	112	53.08
Female	99	46.92
Age, year		
18–29	55	26.07
30–44	79	37.44
45–59	77	36.49
Education		
Junior high school or below	45	21.33
Senior high school	64	30.33
College	67	31.75
Master or above	35	16.59
Individual income, CNY/year		
≤30,000	27	12.80
30,000–80,000	72	34.12
80,000–150,000	63	29.86
150,000–300,000	42	19.91
>300,000	7	3.32
Insurance		
Urban Employees Basic Medical Insurance	143	67.77
Urban And Rural Residents Basic Medical Insurance	65	30.81
Others	3	1.42
City		
Nanjing	75	35.55
Zhengzhou	69	32.70
Xi’an	67	31.75

Abbreviations: CNY, Chinese Yuan. 1 US dollar = 6.73 CNY in 2022.

### 3.2 Preferences estimated using main effects model

All five attributes significantly influenced patients’ choices (*p* < 0.05), as detailed in Table 4. As expected, patients favored more generics passing GCE, generics being reimbursed at a higher rate than brand-name drugs, and greater information disclosure. Interestingly, patients showed a slight preference for prioritizing the use of generics over autonomous choice (0.32 > 0.27) and preferred post-marketing surveillance for certain generics rather than all generics (0.34 > 0.29). However, these differences were not statistically significant (*p* = 0.744 and 0.834) when autonomy and surveillance for all generics were set as reference levels (Supplementary Table S5). In other words, only mandatory substitution and the absence of post-market surveillance significantly negatively impacted patient utility, while the other two levels (autonomy or priority and surveillance of all or some generics) did not significantly affect patient preference. Additionally, there was heterogeneity in preferences across four levels (*p* < 0.05 for SD).

**TABLE 4:** Preference estimated by mixed logit model with only main effects.

Attribute and level	Coefficient (95% CI)	*p*-value	SD (95% CI)	SD *p*-value
Generic consistency evaluation (ref: none)				
Some	1.36 (1.03, 1.70)	<0.001	0.00 (−0.41, 0.41)	0.998
All	2.04 (1.65, 2.42)	<0.001	1.25 (0.90, 1.60)	<0.001
Reimbursement rate (ref: same)				
5% higher	0.34 (0.08, 0.59)	0.010	0.01 (−0.55, 0.56)	0.985
10% higher	0.45 (0.15, 0.75)	0.003	0.66 (0.34, 0.99)	<0.001
Medicine use control (ref: mandatory)				
Priority	0.32 (0.07, 0.56)	0.012	0.34 (−0.13, 0.81)	0.155
Autonomy	0.27 (0.05, 0.49)	0.017	0.51 (0.10, 0.93)	0.014
Information disclosure (ref: no)				
Yes	0.45 (0.22, 0.67)	<0.001	0.60 (0.34, 0.85)	<0.001
Post-marketing surveillance (ref: none)				
Some	0.34 (0.11, 0.57)	0.004	0.04 (−0.38, 0.45)	0.868
All	0.29 (0.10, 0.49)	0.003	0.21 (−0.34, 0.76)	0.454
ASC	0.34 (0.00, 0.67)	0.047	NA	NA
Model specification				
Log likelihood	−851.63			
AIC	1741.27			
BIC	1856.62			

Abbreviations: ASC, alternative-specific constant; AIC, akaike information criterion; BIC, bayesian information criterion.

**FIGURE 1:**
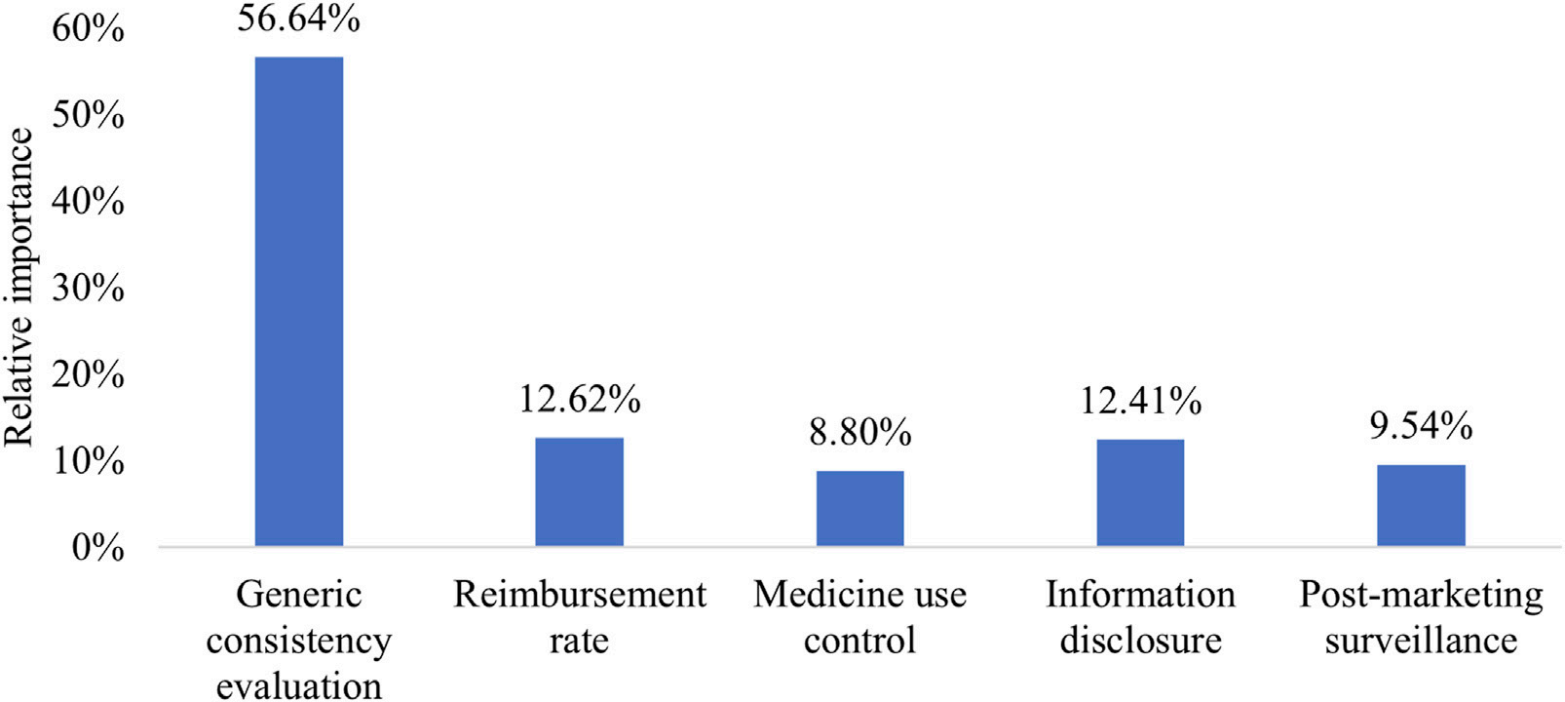
Relative importance of attributes.

Patients perceived the implementation of GCE as significantly more important (RI = 56.64%) compared to other attributes, such as reimbursement rate (RI = 12.62%), information disclosure (RI = 12.41%), post-marketing surveillance (RI = 9.54%), and medicine use control (RI = 8.80%). The importance of GCE was over four times greater than the other attributes (Figure 1).

### 3.3 Preferences heterogeneity

We identified four significant interaction terms, with gender and income being the primary sources of heterogeneity in preferences (Table 5). Males showed a stronger preference for a 10% higher reimbursement rate compared to females (β = 0.53, *p* = 0.008). While females were more likely to choose post-marketing surveillance, both for specific generics (β = −0.45, *p* = 0.044) and for all generics (β = −0.46, *p* = 0.019). As income decreases, there was a greater emphasis on a 5% higher reimbursement rate for generics (β = −0.26, *p* = 0.015). Additionally, other patient characteristics such as age, education, insurance, and city did not significantly affect preference.

**TABLE 5:** Preference estimated by mixed logit model with main effects and interactions.

Attribute and level	Coefficient (95% CI)	*p*-value
Generic consistency evaluation (ref: none)		
Some	1.38 (1.03, 1.72)	<0.001
All	1.83 (1.38, 2.29)	<0.001
Reimbursement rate (ref: same)		
5% higher	−0.49 (−1.21, 0.22)	0.177
10% higher	−0.38 (−1.14, 0.37)	0.322
Medicine use control (ref: mandatory)		
Priority	0.34 (0.08, 0.59)	0.009
Autonomy	0.27 (0.04, 0.49)	0.019
Information disclosure (ref: no)		
Yes	0.44 (0.21, 0.67)	<0.001
Post-marketing surveillance (ref: none)		
Some	0.59 (0.26, 0.93)	<0.001
All	0.52 (0.23, 0.81)	<0.001
ASC	0.35 (0.02, 0.69)	0.040
Interaction terms		
Male* reimbursement rate (10% higher)	0.53 (0.14, 0.91)	0.008
Male* post-marketing surveillance (some)	−0.45 (−0.89, −0.01)	0.044
Male* post-marketing surveillance (all)	−0.46 (−0.85, −0.08)	0.019
Income* reimbursement rate (5% higher)	−0.26 (−0.46, −0.05)	0.015
Model specification		
Log likelihood	−840.1225	
AIC	1730.245	
BIC	1882.018	

Abbreviations: ASC, alternative-specific constant; AIC, akaike information criterion; BIC, Bayesian information criterion.

### 3.4 Uptake rates of policy scenarios

The patient uptake rate of China’s current generic drug policy was only 68.56% (Base scenario). Changing the current policy to mandatory substitution (Scenario 1) decreased the uptake rate of the polices to 59.24%, while other adjustments to the *status quo* policy increased the uptake rate. If GCE is continued until all generics pass while maintaining the *status quo* for other attributes, the uptake rate could reach as high as 82.63% (Scenario 2). Changing the reimbursement rate for generics from the current level to a 10% higher rate while maintaining other attributes at the *status quo* would result in an uptake rate of 78.81% (Scenario 3). Implementing information disclosure could lead to an uptake rate of 78.67% (Scenario 4), similar to the uptake rate with a 10% higher reimbursement rate for generics. Combining the most favorable levels of the five attributes, including all generics passing GCE, a 10% higher reimbursement rate for generics, priority use of generics, information disclosure, and post-marketing surveillance for some generics, resulted in an uptake rate of 94.66% (Scenario 10).

Mandatory substitution has been widely implemented in several countries to promote the use of generics. However, our study substantiated that patient view mandatory substitution as unfavorable. Therefore, we also assessed the uptake rate when mandatory substitution was implemented alongside other policies. The uptake rate reached 76.92% when mandatory substitution was implemented after all generics passed the GCE (Scenario 5). Adding post-marketing surveillance for certain generic drugs to Scenario 5 increased the uptake rate to 83.05% (Scenario 6). The inclusion of either a 10% higher reimbursement rate for generic drugs or information disclosure to Scenario 6 resulted in a further increase in the uptake rate to 88.89% (Scenario 7) or 88.81% (Scenario 8). Implementing mandatory substitution with the most favorable levels of the other four attributes, including all generics passing GCE, a 10% higher reimbursement for generics, information disclosure, and surveillance for some generics, yielded an uptake rate of 92.74% (Scenario 9). Additional scenario analysis details can be found in Table 6.

**TABLE 6:** Estimated uptake rate of hypothetical scenarios.

	Generic consistency evaluation	Reimbursement rate	Medicine use control	Disclosure	Post-marketing surveillance	Uptake rate (95%CI)
Base scenario	Some	Same	Priority	No	None	68.56% (58.86%, 78.26%)
Scenario 1	Some	Same	Mandatory	No	None	59.24% (48.33%, 70.15%)
Scenario 2	All	Same	Priority	No	None	82.63% (75.27%, 89.99%)
Scenario 3	Some	10% higher	Priority	No	None	78.81% (69.39%, 88.23%)
Scenario 4	Some	Same	Priority	Yes	None	78.67% (70.36%, 86.97%)
Scenario 5	All	Same	Mandatory	No	None	76.92% (69.00%, 84.83%)
Scenario 6	All	Same	Mandatory	No	Some	83.05% (75.57%, 90.53%)
Scenario 7	All	10% higher	Mandatory	No	Some	88.89% (82.85%, 94.93%)
Scenario 8	All	Same	Mandatory	Yes	Some	88.81% (82.97%, 94.65%)
Scenario 9	All	10% higher	Mandatory	Yes	Some	92.74% (88.01%, 97.48%)
Scenario 10	All	10% higher	Priority	Yes	Some	94.66% (90.47%, 98.85%)

### 3.5 Sensitivity analysis

The sensitivity analysis included data from 242 patients, incorporating questionnaires that did not select the dominant option. Results obtained from a mixed logit model were presented in Supplementary Table S6 and Supplementary Figure S1. Similar to the main analysis, GCE was highly valued (RI = 58.14%). Although there was a change in importance rankings between information disclosure and reimbursement rate, their RIs remained comparable at 12.60% and 11.53%.

## 4 Discussion

This study indicated that patients considered GCE to be the most critical in generic drug policies, followed by reimbursement rate, information disclosure, post-marketing surveillance, and medicine use control. We found that the current policy promoting generic substitution in China could be further optimized to enhance patient acceptance and satisfaction. Accordingly, China should continue implementing the GCE, increase reimbursement rates for generics, and enhance information disclosure. These measures can effectively counterbalance the negative impact of mandatory substitution on patients. Given the importance of patients’ perspectives on generic substitution, our findings provide valuable insights for policymakers in China and other countries to optimize generic drug policies and promote generic substitution.

Two new attributes, GCE and post-marketing surveillance, were included based on Chinese policy and patient concerns identified in focus group discussions. These two attributes have not previously been reported. This discrepancy may be attributed to the omission of the most recent policies and the lack of focus on China’s generic substitution policies in previous studies. Moreover, focus group discussions permit a comprehensive understanding of the concerns of the target group within a specific cultural or social context. The decision to exclude an opt-out option was made after careful consideration of the research objective and the desire to simplify the decision-making process for respondents. However, without the opt-out option, respondents were forced to choose between the available alternatives, potentially deviating from their true preferences.

Our study found that GCE significantly influenced patients’ acceptance of generic promotion policies. GCE is a recently implemented policy in China that evaluates the bioequivalence of generic drugs to their original counterparts. Bioequivalence with reference drugs is the basis for approving generic drugs. Previously, China did not limit the choice of reference formulations to the original drug, allowing selection from other available generic drugs in the market. Similar to our findings, previous research has highlighted the significance of persuading physicians, pharmacists, and patients regarding the bioequivalence of generics to original drugs (Wouters et al., 2017). Consequently, it is imperative for China to maintain the implementation of GCE to improve the quality of generic drugs.

While our study revealed that patients favored higher reimbursement rates for generic drugs, previous empirical research has demonstrated that merely reducing copays for generics and increasing copays for brand-name drugs did not result in a shift toward generics (Rodin et al., 2009; Sen et al., 2014). This inconsistency can be attributed to the differences between stated and revealed preferences. Although patients may indicate a preference for generic drugs when they receive higher reimbursements, they may consider other factors, such as effectiveness and safety, when making the actual choice between generic and brand-name drugs. Additionally, the preference for increased reimbursement rates for generic medications varied based on the patient’s gender and income. Thus, it may be necessary to create diverse reimbursement programs that consider the patient’s circumstances.

A previous systematic review revealed that providing patients with information that dispels misconceptions about generics can enhance confidence in generic medicines (Dunne, 2016), consistent with our research and indicating the necessity of information disclosure. Our study also revealed that post-marketing surveillance ranked fourth in importance among the five attributes considered, and females expressed a higher level of concern than males. Notably, there were no significant differences observed among patients regarding post-marketing surveillance for all or some generics. This suggests that patients perceive monitoring all generics as unnecessary and that surveillance efforts should be focused on those with high risks.

Mandatory substitution has been widely adopted to promote the use of generic drugs, although it has not yet been implemented in China. Our findings revealed that mandatory substitution is detrimental to patient acceptance of the policy. Nevertheless, it is worth noting that mandatory substitution ranked last in terms of importance among the five attributes, indicating mild dissatisfaction among respondents.

Heterogeneity in preferences based on gender and income was observed. Gender differences in preferences for reimbursement rates and post-marketing surveillance were evident. This variation between males and females may be attributed to the influence of social norms and cultural expectations on health-related preferences, including economic considerations and risk perceptions. Previous studies have also highlighted gender disparities in preferences (Iglesias Urrutia et al., 2022; Ding et al., 2024).

Low uptake rates in the current situation indicate the need for policy adjustments to better align with patient expectations and needs. Based on the analysis of uptake rates in different scenarios, GCE was most likely to lead to high levels of uptake and successful policy acceptance. Therefore, it is recommended that policymakers prioritize the implementation of GCE until all generic drugs passed it. Our study revealed that information disclosure significantly influenced the uptake rate of generics policy, comparable to the impact of a 10% increase in the reimbursement rate and the implementation of post-marketing surveillance for generics. However, information disclosure appears to be a more feasible and cost-effective approach compared to the latter two. In addition, we observed a higher uptake rate of mandatory substitution when combined with other policies, such as the requirement for all generics to pass GCE, compared to the current situation. This implies that patients would not be concerned about mandatory substitution and the resulting loss of autonomy if a generic drug can demonstrate bioequivalence and interchangeability with the originator.

There were several limitations in this study. Firstly, the small sample size and sampling of patients from only three hospitals may limit the generalizability of this study. Indeed, increasing the sample size in future studies could yield more robust and generalizable findings. Secondly, this study only included the five policy attributes that were considered most important, potentially neglecting other significant attributes. However, including too many attributes in a DCE can overwhelm respondents cognitively (Soekhai et al., 2019). Thirdly, the attribute selection, informed by expert opinion, may carry the risk of introducing bias, as experts might prioritize certain attributes aligned with their own professional experiences. There is a possibility that attributes excluded from the final DCE could still play a significant role in the patient’s decision-making process. Fourthly, our study only elicited the preferences of young adult patients. The preferences of elderly patients were not included in our analysis, which may affect the representativeness and generalizability of our results. Although those older than 60 years might constitute the majority of generic drug users, we found that patients over 60 years old had difficulty understanding and completing the DCE during the pre-survey. Consequently, they were not included in the formal survey. Lastly, this study did not investigate physician preferences despite the significant role they play in making prescribing decisions. There is ongoing research on physician preferences for generics policy, and the findings will be presented in a separate publication.

## 5 Conclusion

China’s GCE policy significantly influenced patient preferences. To enhance patient acceptance, it is recommended to maintain the implementation of GCE, raise reimbursement rates for generic drugs, and improve information disclosure. Furthermore, policies should be customized based on individual patient circumstances, such as gender and income.

## Data Availability

The raw data supporting the conclusions of this article will be made available by the authors, without undue reservation.

## References

[B1] Babar Z. U. D. Kan S. W. Scahill S. (2014). Interventions promoting the acceptance and uptake of generic medicines: a narrative review of the literature. Health Policy 117, 285–296. 10.1016/j.healthpol.2014.06.004 24973926

[B2] Bridges J. F. P. Hauber A. B. Marshall D. Lloyd A. Prosser L. A. Regier D. A. (2011). Conjoint analysis applications in health—a checklist: a report of the ISPOR good research practices for conjoint analysis task force. Value Health 14, 403–413. 10.1016/j.jval.2010.11.013 21669364

[B3] Campbell D. Erdem S. (2019). Including opt-out options in discrete choice experiments: issues to consider. Patient 12, 1–14. 10.1007/s40271-018-0324-6 30073482

[B4] Chandoevwit W. Wasi N. (2020). Incorporating discrete choice experiments into policy decisions: case of designing public long-term care insurance. Soc. Sci. Med. 258, 113044. 10.1016/j.socscimed.2020.113044 32497823

[B5] Choudhry N. K. Denberg T. D. Qaseem A. Clinical Guidelines Committee of American College of Physicians (2016). Improving adherence to therapy and clinical outcomes while containing costs: opportunities from the greater use of generic medications: best practice advice from the clinical guidelines committee of the American college of physicians. Ann. Intern Med. 164, 41–49. 10.7326/M14-2427 26594818

[B6] Conrad R. Davis K. Glos L. Liu W. (2022). Estimating cost savings from new generic drug approvals in 2018-2020. Available at: https://www.fda.gov/media/161540/download.

[B7] Ding R. Shao R. Zhang L. Yan J. (2024). Preferences and willingness to pay for medication in patients with renal cell carcinoma in China: a discrete-choice experiment. Patient 17, 97–108. 10.1007/s40271-023-00659-2 38030868

[B8] Di Paolo A. Arrigoni E. (2018). Generic substitution of orphan drugs for the treatment of rare diseases: exploring the potential challenges. Drugs 78, 399–410. 10.1007/s40265-018-0882-x 29464665

[B9] Dunne S. S. (2016). What do users of generic medicines think of them? a systematic review of consumers’ and patients’ perceptions of, and experiences with, generic medicines. Patient 9, 499–510. 10.1007/s40271-016-0176-x 27142371

[B10] El-Dahiyat F. Kayyali R. (2013). Evaluating patients’ perceptions regarding generic medicines in Jordan. J Pharm Policy Pract 6, 3–8. 10.1186/2052-3211-6-3 24764538 PMC3987061

[B11] El-Harakeh A. Haley S. J. (2022). Improving the availability of prescription drugs in Lebanon: a critical analysis of alternative policy options. Health Res. Policy Sys 20, 106. 10.1186/s12961-022-00921-3 PMC9547632 36209085

[B12] General Office of the State Council of China (2016). Opinions on the evaluation of the consistency of quality and efficacy of generic drugs. Available at: https://www.gov.cn/zhengce/content/2016-03/05/content_5049364.htm.

[B13] Geng J. Bao H. Feng Z. Meng J. Yu X. Yu H. (2022). Investigating patients’ preferences for new anti-diabetic drugs to inform public health insurance coverage decisions: a discrete choice experiment in China. BMC Public Health 22, 1860. 10.1186/s12889-022-14244-z 36199056 PMC9533494

[B14] Granlund D. Sundström D. (2018). Physicians prescribing originals causes welfare losses. Econ. Lett. 170, 143–146. 10.1016/j.econlet.2018.06.017

[B15] Hassali M. A. Alrasheedy A. A. McLachlan A. Nguyen T. A. Al-Tamimi S. K. Ibrahim M. I. M. (2014). The experiences of implementing generic medicine policy in eight countries: a review and recommendations for a successful promotion of generic medicine use. Saudi Pharm. J. 22, 491–503. 10.1016/j.jsps.2013.12.017 25561861 PMC4281627

[B16] Howard J. N. Harris I. Frank G. Kiptanui Z. Qian J. Hansen R. (2018). Influencers of generic drug utilization: a systematic review. Res. Soc. Adm. Pharm. 14, 619–627. 10.1016/j.sapharm.2017.08.001 PMC5910277 28814375

[B17] Iglesias Urrutia C. P. Erdem S. Birks Y. F. Taylor S. J. C. Richardson G. Bower P. (2022). People’s preferences for self-management support. Health Serv. Res. 57, 91–101. 10.1111/1475-6773.13635 33634466 PMC8763292

[B18] Kaplan W. A. Ritz L. S. Vitello M. Wirtz V. J. (2012). Policies to promote use of generic medicines in low and middle income countries: a review of published literature, 2000–2010. Health Policy 106, 211–224. 10.1016/j.healthpol.2012.04.015 22694970

[B19] Lancsar E. Louviere J. (2008). Conducting discrete choice experiments to inform healthcare decision making: a user’s guide. Pharmacoeconomics 26, 661–677. 10.2165/00019053-200826080-00004 18620460

[B20] Lancsar E. Louviere J. Flynn T. (2007). Several methods to investigate relative attribute impact in stated preference experiments. Soc. Sci. Med. 64, 1738–1753. 10.1016/j.socscimed.2006.12.007 17257725

[B21] Mouter N. Boxebeld S. Kessels R. van Wijhe M. de Wit A. Lambooij M. (2022). Public preferences for policies to promote COVID-19 vaccination uptake: a discrete choice experiment in The Netherlands. Value Health 25, 1290–1297. 10.1016/j.jval.2022.03.013 35527162 PMC9069307

[B22] Nicolet A. Perraudin C. Wagner J. Gilles I. Krucien N. Peytremann-Bridevaux I. (2022). Patient and public preferences for coordinated care in Switzerland: development of a discrete choice experiment. Patient 15, 485–496. 10.1007/s40271-021-00568-2 35067858 PMC9197802

[B23] Obadha M. Chuma J. Kazungu J. Abiiro G. A. Beck M. J. Barasa E. (2020). Preferences of healthcare providers for capitation payment in Kenya: a discrete choice experiment. Health Policy Plan. 35, 842–854. 10.1093/heapol/czaa016 32537642 PMC7487334

[B24] Rodin H. A. Heaton A. H. Wilson A. R. Garrett N. A. Plocher D. W. (2009). Plan designs that encourage the use of generic drugs over brand-name drugs: an analysis of a free generic benefit. Am. J. Manag. Care 15, 881–888. 20001169

[B25] Sacks C. A. Kesselheim A. S. Sarpatwari A. Patel L. (2021). Assessment of variation in state regulation of generic drug and interchangeable biologic substitutions. JAMA Intern. Med. 181, 16–22. 10.1001/jamainternmed.2020.3588 32865564 PMC7489381

[B26] Sen B. Blackburn J. Morrisey M. Becker D. Kilgore M. Caldwell C. (2014). Can increases in CHIP copayments reduce program expenditures on prescription drugs? Medicare Medicaid Res. Rev. 4. 10.5600/mmrr2014-004-02-a03 PMC4063370 24967148

[B27] Soekhai V. de Bekker-Grob E. W. Ellis A. R. Vass C. M. (2019). Discrete choice experiments in health economics: past, present and future. Pharmacoeconomics 37, 201–226. 10.1007/s40273-018-0734-2 30392040 PMC6386055

[B28] Sun J. (2013). International experiences of promoting generics use and its implications to China. J. Evid. Based Med. 6, 74–80. 10.1111/jebm.12030 23829799

[B29] Veldwijk J. Lambooij M. S. de Bekker-Grob E. W. Smit H. A. de Wit G. A. (2014). The effect of including an opt-out option in discrete choice experiments. PLoS One 9, e111805. 10.1371/journal.pone.0111805 25365169 PMC4218820

[B30] Wouters O. J. Kanavos P. G. McKEE M. (2017). Comparing generic drug markets in Europe and the United States: prices, volumes, and spending. Milbank Q. 95, 554–601. 10.1111/1468-0009.12279 28895227 PMC5594322

[B31] Yuan J. Lu Z. K. Xiong X. Jiang B. (2021). Lowering drug prices and enhancing pharmaceutical affordability: an analysis of the national volume-based procurement (NVBP) effect in China. BMJ Glob. Health 6, e005519. 10.1136/bmjgh-2021-005519 PMC8438819 34518200

[B32] Zhang C. Ding Y. Wu Z. Wang J. Wu X. Xie W. (2023). Does China’s competitive generic substitution policy deliver equivalent clinical outcomes? A pilot study with two generic formulations of olanzepine. Front. Pharmacol. 14, 1097600. 10.3389/fphar.2023.1097600 36909190 PMC9999380

[B33] Zhao M. Zhang L. Feng Z. Fang Y. (2021). Physicians’ knowledge, attitude and practice of generic substitution in China: a cross-sectional online survey. Int. J. Environ. Res. Public Health 18, 7749. 10.3390/ijerph18157749 34360043 PMC8345361

