# Supplementary material for: Patient preferences for generic substitution policies: a discrete choice experiment in China
Source: Front Pharmacol. 2024 Jul 2;15:1400156. doi: 10.3389/fphar.2024.1400156 (PMC11250648; doi:10.3389/fphar.2024.1400156)
Supplement: Supplementary file 1 [file Table1.DOCX]

Supplementary Material

**Table S1** Candidate attributes form literature review.

|  | **Candidate attributes** |
| --- | --- |
| 1 | Pricing |
| 2 | Generic prescribing |
| 3 | Mandatory generic substitution |
| 4 | Patents |
| 5 | Reimbursement |
| 6 | Education |
| 7 | Medicines use review |
| 8 | Advertisement |

**Table S2** Key opinions during the expert interview.

|  | **Key opinions** |
| --- | --- |
| Expert A | Modifying the prescription system is a huge challenge in China. This should not be included in generic drug substitution policies. |
| Expert B | Attributes such as "patent" that were difficult for patients to understand. |
| Expert C | "Education" and "advertisement" in Chinese can easily cause misunderstandings among patients. Combine "education" and "advertisement" into one attribute, information disclosure, for greater clarity and conciseness. |
| Expert D | Incorporating a diverse range of policy instruments to avoid attribute homogeneity. I suggested that you should include regulatory, economic and informational instruments among the attributes according to Vedung E's categorization of public policy. For example, both "reimbursement" and "pricing" were economic means. |
| Expert E | The government has already abolished drug pricing, and I do not believe that pricing should be included as an attribute. |
| Expert F | Medicines use review in China is primarily used to ensure rational drug use. Currently, the review does not include generic drug substitution, which can easily lead to patient misunderstandings. |
| Expert G | We have not implemented mandatory substitution; we only prioritize the use of drugs that have won bids in NVBP. Therefore, it should not be set as a matter of whether there is mandatory substitution or not, but rather should include three levels. |

**Table S3** Prior parameters in the D-efficient design.

| **Levels** | **Prior parameters** |
| --- | --- |
| Generic consistency evaluation (some) | 0.001 |
| Generic consistency evaluation (all) | 0.002 |
| Reimbursement rate (5% higher) | 0.001 |
| Reimbursement rate (10% higher) | 0.002 |
| Medicine use control (priority) | 0.001 |
| Medicine use control (autonomy) | 0.002 |
| Information disclosure (yes) | 0.001 |
| Post-marketing surveillance (some) | 0.001 |
| Post-marketing surveillance (all) | 0.002 |

**Table S4** Quotas and sample.

| **Characteristics** | | **Quotas*** | **Completed the questionnaire (N=302)** | | **P-value** |
| --- | --- | --- | --- | --- | --- |
|  |  | % | % | N |  |
| Gender | Male | 51.17% | 52.98% | 160 | 0.777 |
|  | Female | 48.83% | 47.02% | 142 |  |
| Age | 18-29 | 23.08% | 24.17% | 73 | 0.983 |
|  | 30-44 | 37.24% | 37.09% | 112 |  |
|  | 45-59 | 39.68% | 38.74% | 117 |  |
| Department | Inpatient | 50% | 50.66% | 153 | 0.888 |
|  | Outpatient | 50% | 49.34% | 149 |  |
| Department | Internal medicine | 25% | 26.82% | 81 | 0.984 |
|  | Surgery | 25% | 25.17% | 76 |  |
|  | Obstetrics/Gynaecology | 25% | 24.83% | 75 |  |
|  | Others (e.g., Pentacenter, Dermatology) | 25% | 23.18% | 70 |  |

* The quotas of gender and age was determined based on the 2020 China population census, which can be accessed at: http://www.stats.gov.cn/sj/pcsj/rkpc/7rp/zk/indexch.htm.

**Table S5** Preference weights when the reference level changes.

| **Attribute and level** | **Coefficient (95% CI)** | **P-value** | **SD (95% CI)** | **SD P-value** |
| --- | --- | --- | --- | --- |
| Generic consistency evaluation (ref: none) | | | | |
| Some | 1.39 (1.05, 1.73) | < 0.001 | -0.01 (-0.43, 0.41) | 0.958 |
| All | 2.11 (1.70, 2.51) | < 0.001 | 1.33 (0.95, 1.72) | < 0.001 |
| Reimbursement rate (ref: same) | | | | |
| 5% higher | 0.42 (0.16, 0.69) | 0.002 | 0.05 (-0.75, 0.85) | 0.904 |
| 10% higher | 0.49 (0.18, 0.80) | 0.002 | 0.70 (0.37, 1.03) | < 0.001 |
| Medicine use control (ref: autonomy) | | | | |
| Priority | 0.04 (-0.22, 0.30) | 0.744 | 0.15 (-0.61, 0.92) | 0.692 |
| Mandatory | -0.28 (-0.52, -0.04) | 0.024 | 0.82 (0.51, 1.14) | < 0.001 |
| Information disclosure (ref: no) | | | | |
| Yes | 0.46 (0.23, 0.70) | < 0.001 | 0.60 (0.33, 0.88) | < 0.001 |
| Post-marketing surveillance (ref: all) | | | | |
| Some | 0.02 (-0.18, 0.23) | 0.834 | 0.01 (-0.47, 0.49) | 0.971 |
| None | -0.31 (-0.51, -0.11) | 0.003 | -0.24 (-0.95, 0.47) | 0.510 |
| ASC | 0.35 (0.01, 0.69) | 0.044 | NA | NA |
| Model specification | | | | |
| Log likelihood | -845.67 |  |  |  |
| AIC | 1729.34 |  |  |  |
| BIC | 1844.68 |  |  |  |

ASC, alternative-specific constant; AIC, Akaike information criterion; BIC, Bayesian information criterion.

**Table S6** Preference weights including patients who did not pass the quality control test.

| **Attribute and level** | **Coefficient (95% CI)** | **P-value** | **SD (95% CI)** | **SD P-value** |
| --- | --- | --- | --- | --- |
| Generic consistency evaluation (ref: none) | | | | |
| Some | 1.42 (1.12, 1.72) | < 0.001 | 0.02 (-0.38, 0.41) | 0.933 |
| All | 2.11 (1.76, 2.45) | < 0.001 | 1.16 (0.85, 1.46) | < 0.001 |
| Reimbursement rate (ref: same) | | | | |
| 5% higher | 0.25 (0.01, 0.48) | 0.037 | 0.00 (-0.41, 0.40) | 0.998 |
| 10% higher | 0.42 (0.14, 0.69) | 0.003 | 0.60 (0.31, 0.90) | < 0.001 |
| Medicine use control (ref: mandatory) | | | | |
| Priority | 0.32 (0.09, 0.54) | 0.006 | 0.33 (-0.11, 0.76) | 0.141 |
| Autonomy | 0.32 (0.11, 0.52) | 0.002 | 0.56 (0.19, 0.93) | 0.003 |
| Information disclosure (ref: no) | | | | |
| Yes | 0.46 (0.25, 0.67) | < 0.001 | 0.66 (0.44, 0.88) | < 0.001 |
| Post-marketing surveillance (ref: none) | | | | |
| Some | 0.32 (0.11, 0.53) | 0.003 | 0.04 (-0.35, 0.43) | 0.832 |
| All | 0.28 (0.11, 0.46) | 0.002 | 0.09 (-0.52, 0.70) | 0.776 |
| ASC | 0.29 (-0.01, 0.60) | 0.060 | NA | NA |
| Model specification | | | | |
| Log likelihood | -1017.976 |  |  |  |
| AIC | 2073.951 |  |  |  |
| BIC | 2192.92 |  |  |  |

ASC, alternative-specific constant; AIC, Akaike information criterion; BIC, Bayesian information criterion.

**Figure S1** Relative importance of attributes including patients who did not pass the quality control test.
